# Identifying state-level policy and provision domains for physical education and physical activity in high school

**DOI:** 10.1186/1479-5868-10-86

**Published:** 2013-07-01

**Authors:** Derek Hales, June Stevens, David M Murray, Dan R Taber, Amy Roberts

**Affiliations:** 1 Center for Health Promotion and Disease Prevention, University of North Carolina at Chapel Hill, 1700 Martin Luther King Jr. BLVD. CB#7426, Chapel Hill, NC 27599, USA; 2 Department of Nutrition, University of North Carolina at Chapel Hill, 245 Rosenau Hall CB#7461, Chapel Hill, NC 27599, USA; 3 Biostatistics and Bioinformatics Branch Division of Epidemiology, Statistics, and Prevention Research Eunice Kennedy Shriver National Institute of Child Health and Human Development National Institutes of Health, 6100 Executive Boulevard, 2B03, Rockville, MD 20892, USA; 4 Institute for Health Research and Policy, University of Illinois at Chicago (MC 275), 432 Westside Research Office Bldg, 1747 West Roosevelt Road, Chicago, IL 60608, USA

**Keywords:** Policy, Physical Activity, Physical Education, SHPPS

## Abstract

**Background:**

It is important to quickly and efficiently identify policies that are effective at changing behavior; therefore, we must be able to quantify and evaluate the effect of those policies and of changes to those policies. The purpose of this study was to develop state-level physical education (PE) and physical activity (PA) policy domain scores at the high-school level. Policy domain scores were developed with a focus on measuring policy change.

**Methods:**

Exploratory factor analysis was used to group items from the state-level School Health Policies and Programs Study (SHPPS) into policy domains. Items that related to PA or PE at the High School level were identified from the 7 SHPPS health program surveys. Data from 2000 and 2006 were used in the factor analysis. RESULTS: From the 98 items identified, 17 policy domains were extracted. Average policy domain change scores were positive for 12 policy domains, with the largest increases for “Discouraging PA as Punishment”, “Collaboration”, and “Staff Development Opportunities”. On average, states increased scores in 4.94 ± 2.76 policy domains, decreased in 3.53 ± 2.03, and had no change in 7.69 ± 2.09 policy domains. Significant correlations were found between several policy domain scores.

**Conclusions:**

Quantifying policy change and its impact is integral to the policy making and revision process. Our results build on previous research offering a way to examine changes in state-level policies related to PE and PA of high-school students and the faculty and staff who serve them. This work provides methods for combining state-level policies relevant to PE or PA in youth for studies of their impact.

## Background

In the United States, state and local governments have far-reaching responsibilities for public schools and the youth attending those schools, including their health and welfare. In recent years growing concerns about the epidemic of childhood obesity and low levels of physical activity (PA) have prompted the establishment of a large number of legislative and regulatory actions that aim to, directly or indirectly, increase PA in schools. In 2011, 41 states and the District of Columbia (DC) had legislation introduced that was related to PA or Physical Education (PE) in schools *(Database of State Legislative and Regulatory Action to Prevent Obesity and Improve Nutrition and Physical Activity, accessed Jan 2012).* While previous research has shown that some state-level legislation and local policies are positively related to PE time and PA levels of students [1-5] there is little empirical support for many of the legislative actions that are pending or have been enacted. This includes support for legislative action directly related to PA (e.g. allowing community access to school playgrounds and field) and legislation more peripheral to PA levels (e.g. creating a model framework for teacher and principal evaluation instruments or requiring public meetings about education issues). Without evidence for effectiveness it is not known which policy actions are useful and which are ineffectual, placing an undue burden on a system with limited resources.

As state budgets tighten, it becomes increasingly important to quickly and efficiently identify policies that are effective. This requires methods to quantify policies and policy change in a meaningful way to allow careful evaluation of implemented policies. This measurement task is difficult due to the large numbers and types of policies, many of which are strongly related to each other in terms of their specific goal, target behavior and/or agent of change. While policies can be evaluated one-by-one, it seems obvious that related policies will interact with each other in real life settings and that examining each policy individually could yield misleading results. Indeed, previous research in this area has suggested that due to the complexity and reach of state-level legislation it may be more effective to evaluate changes in policy factors or domains defined as combinations of individual policies that may overlap and tend to change and act together [6].

We are aware of two systems or policy scoring mechanisms that have been developed to group and quantify school-level PA and/or PE policies [6,7]. One of these, the Physical Education and Recess State Policy Classification System (PERSPCS), was developed to access the “nature and extent” of state-level PE statutes and regulations in six areas: PE time, PA time, staffing, curriculum, assessment and recess [7]. The system uses a rating scale (e.g. 0 to 4) that allows each policy area to be “graded” based on the strength, specificity and comprehensiveness of the legislation. Summary and area specific (e.g. PE Time, curriculum) scores can be computed for elementary, middle, and high school levels and for all grade levels combined. Currently, state-level ratings are available from 2003 to 2008 and 2010. While the development of this system was an important move forward, it is somewhat limited in scope, covering only a few policy domains, and may require specialized legal training to grade policy areas accurately.

A second policy scoring system was developed as a comprehensive measure of state-level, school-based obesity prevention policies using data collected as part of the 2006 School Health Policies and Programs Study (SHPPS; [6]). At the state-level, the purpose of SHPPS is to provide data that can be used to describe policies and programs from seven school health program components. Nanney et al. created a PA policy scoring system using 146 items from the PA and PE components of SHPPS. These items were grouped into 10 policy domains using principal components factor analysis, expert opinion, and the relationships among items and policy domains. This approach capitalized on the large number of policy and provision items to construct policy domain scores that combined multiple items to create robust measures of important policy areas. Policy domain specific and an overall summary score were computed using the proportion of policies characterized as “required” (score = 1). Despite several strengths, the system lacks grade specific policy domain scores, which are useful because PE requirements and implementation are different across grade levels. In addition the policy domain scores were developed using only items from the 2006 version of the SHPPS survey, making it difficult to use them to evaluate the frequency and impact of policy change if item content and response options change from one administration to the next.

In this paper we build upon this previous research to develop state-level high-school PE/PA policy domain scores specifically designed with a focus on policy change. We use information from both the 2000 and 2006 SHPPS surveys to identify the policy domains that can be used to assess change over that period. We describe a set of policy domain scores that can be computed using surveillance data collected as part of the SHPPS survey and present State-level policy domain scores and change. Exploratory factor analysis was used to identify groups of items or variables that were statistically related and together represented a concept or domain of interest. Items that grouped together have shared variance and can be combined, or modeled, as a single variable. This combination of information from multiple related items generally results in more robust variables and simplified statistical models that are representative of the relationships among the individual items but easier to interpret and apply to processes like policy evaluation.

## Methods

### 2000 and 2006 SHPPS data

Data for this study are from the 2000 and 2006 SHPPS [8-11]. This national survey is conducted by the Centers for Disease Control and Prevention every 6 years, and is designed to collect information on school health policies (e.g. Has your state adopted a policy…) and practices (e.g. Has your state provided funding or offered…) at the state, district, school, and classroom levels. For this work we use only state-level data for high schools. Although SHPPS provides data for many grade levels, this analysis was limited to high school to allow for future comparisons with the PA YRBS data, which is only available for high school students.

In the SHPPS survey, “policy” is defined as:

“any law, rule, regulation, administrative order, or similar kind of mandate issued by the state board of education, state legislature, or other state agency with authority over schools in your state.”

SHPPS data were collected through computer-assisted telephone interviews or self-administered mailed questionnaires from state personnel who are considered most knowledgeable about the relevant policy area. In 87% of states, the PE component of the survey was completed by the self-identified coordinator of PE. All states and the District of Columbia (included in the term “states” from here on) participated in SHPPS in both 2000 and 2006.

**Figure 1:**
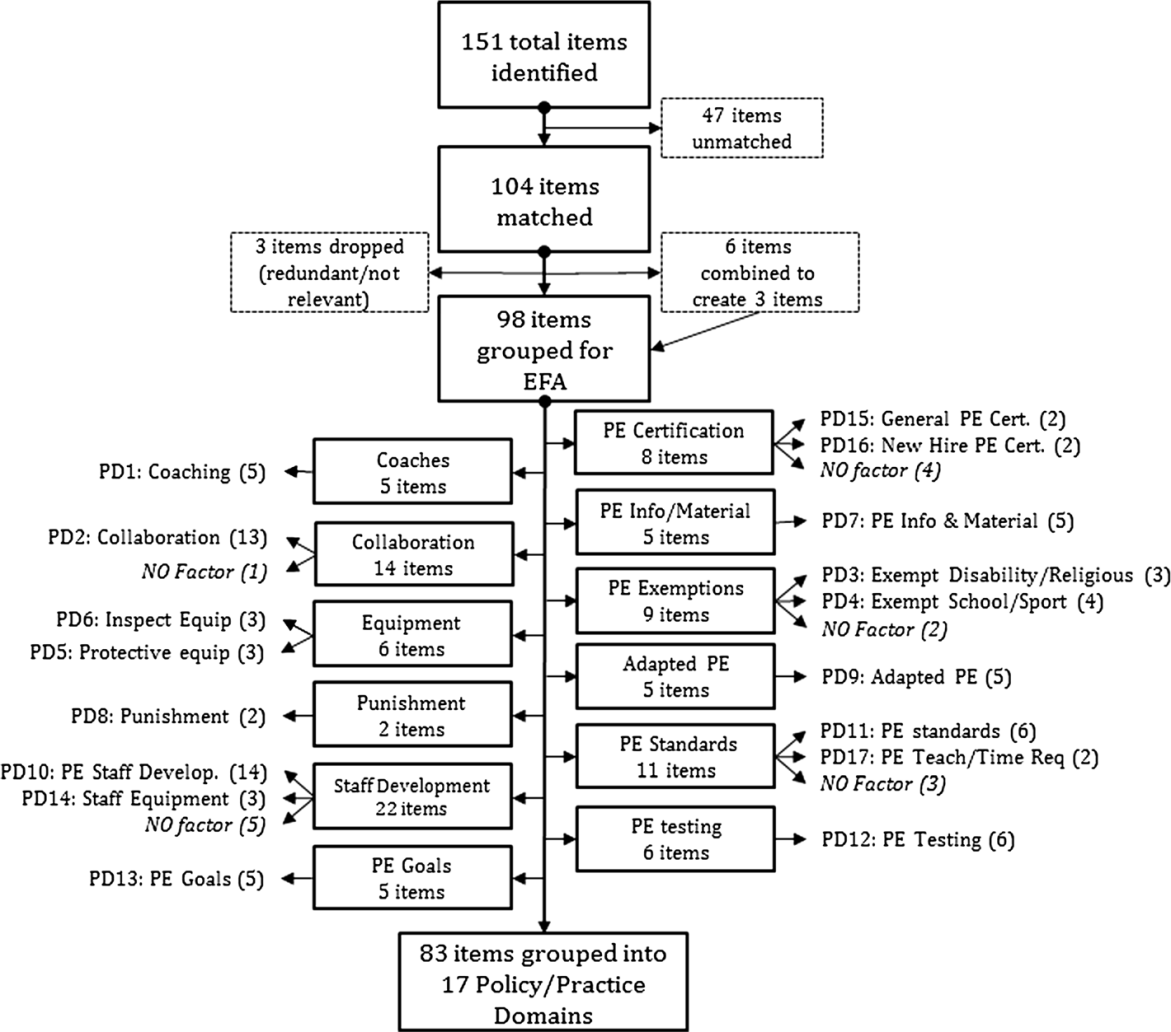
**Summary of item selection, item grouping, and policy domains.** Cert. = Certification; Req. = Requirement; EFA = exploratory factor analysis PD# = Policy Domain Number in ( ) = # of items in policy domain.

SHPPS contains 7 health program component surveys: 1. Faculty and Staff Health Promotion; 2. School Policy and Environment; 3. Food Service; 4. Health Education; 5. Health Services; 6. Mental Health; and Social Services; 7. Physical Education. For this project, items from all 7 surveys were examined to identify questions that related to PA or PE at the high school level. In total, 151 items were identified (see Figure 1). Items were compared between the 2000 and 2006 surveys to ensure that policy domain change scores could be computed. Items were checked for wording (removal or addition of information), format, and response options at both time points. Of the items identified, 104 were sufficiently similar in the 2000 and 2006 surveys that they could be matched for the purpose of calculating change scores. Three of these items were found to have irrelevant or redundant information. In addition, we decided that the information from three pairs of questions (6 items) that were connected through skip patterns should be combined to create 3 items with 3-levels each (NO, Recommend, Require). These questions asked respondents if the state had a written policy about some topic, if they answered “YES” they were classified as having a policy. If they answered “NO” a follow-up question was asked about recommendation of this topic (YES/NO). Responses to the 98 items collected in the 51 states were used for this analysis.

### State-level policy domains

Policy domains were developed using the results from several exploratory factor analysis models, item grouping from the SHPPS survey, and item/scale psychometrics. Analyses were conducted separately for data from the 2000 and 2006 SHPPS using available information from all 51 states. A summary of item selection, item grouping, and the final policy domains can be found in Figure 1.

All items were scored on a two (NO/YES) or three (NO, Recommend, Require) level scale. Details on the SHPPS scoring system are available in the technical documentation for the survey [12]. For the purpose of this project items were scored 0 for no policy or 1 for presence of a policy. Several items included a middle category, recommend/encourage; this was scored 0.5 to simplify the creation of factors. The ratio of the sample (51) to items (98) was small, which could reduce stability of the exploratory factor analysis results [13]. Therefore, we initially grouped items based on the structure of the SHPPS survey and previous research [6]. The grouping resulted in 12 exploratory factor analysis models with sample to item ratios ranging from 2.5:1 to 10:1. It was expected that by increasing this ratio the results of the exploratory factor analysis would be more stable.

One of the goals of this project was to develop policy domains that could be used to examine policy change. To ensure this, decisions about item retention and factor selection were done systematically using both sets of results (2000 and 2006). The final factors from SHPPS 2000 contained the same set of items as the final factors from SHPPS 2006. During this process the exploratory factor analysis for a group of items was conducted in both samples. Results were then compared. Any item with no factor loading (correlation between the factor and the variable) greater than 0.40 in either sample was removed, and the exploratory factor analysis was repeated. The next steps involved identifying individual items that that did not fit well at one of the time points. These items were removed individually with the rule that final factors had to have the same items in both data sets. Most items were excluded due to low factor loadings (< 0.40) or large cross loadings (correlation with another factor) (> 0.40). For several factors, the final models produced estimates with negative error variance for an item. While not ideal, the occurrence of Heywood cases, items with negative variance estimates, is not unexpected given the size of the sample [13]. In each of these cases the final model and items were inspected for over-factoring and relationships among the items were examined using correlations, Cronbach’s alpha, and item-total correlations. All exploratory factor analyses were conducted using a robust weighted least squares estimator (WLSMV), Geomin rotation, and variables classified as categorical. MPLUS v6 was used for these analyses.

### State-level policy domain changes

Summaries and comparisons of policy domain and policy domain change scores were estimated using SAS v9.2. Scores were computed for each policy domain using the 2000 and 2006 data and Cronbach’s alpha was calculated [14]. Policy domain change scores were computed as (score 2006 – score 2000), and were considered “no change” from 2000 to 2006 if the value changed by less than 20% of the policy domain change score standard deviation. Most states with no change had policy domain change scores of 0.

## Results

### State-level policy domains

Based on results from the exploratory factor analysis 17 policy domains were extracted using 83 of the original 98 items selected (Table 1 and Figure 1). Sample sizes for the exploratory factor analyses ranged from 45 to 51 states, with 75% including at least 49 states. Three items did not have any variation in the 2000 sample, but were found to be significant in the model for 2006. These items were retained for their respective policy domain scores. Four of the final policy domains included only 2 items each, while 8 policy domains contain 5 or more items each. A complete list of the items in each policy domain is provided in Additional file 1.

**Table 1:** Summary of policy domains, factor loadings, internal consistency (alpha), policy domain scores (mean, SD), and policy domain change score (mean, SD)

**Policy domain (# items)**	**Example item**	**Factor loadings (min-max)**	**Alpha (2000/ 2006)**	**Policy domain scores**					
				**2000**		**2006**		**Change**	
				**n**	**Mean (SD)**	**n**	**Mean (SD)**	**n**	**Mean (SD)**
Coaching (5)	Has your state adopted a policy stating that head coaches of interscholastic sports will have a teaching certificate? (YES/NO)	0.440 -0.968	0.61/ 0.66	47	0.272 (0.299)	49	0.299 (0.302)	45	0.037 (0.332)
Collaboration (13)	During the past 12 months, have state physical education staff worked on physical education activities with staff or members from state-level health organizations (e.g. AHA, ACS)? (YES/NO)	0.464 - .995	0.89/ 0.86	51	0.545 (0.319)	51	0.703 (0.262)	51	0.158 (0.358)
Exemptions from PE religious or disability (3)	Based on policies adopted by your state, can senior high school students be exempt from physical education requirements for one grading period or longer for religious reasons? (YES/NO)	0.603 – 1.05	0.54/ 0.75	49	0.218 (0.301)	49	0.197 (0.319)	48	−0.021 (0.303)
Exemptions from PE for school or sport participation (4)	Based on policies adopted by your state, can senior high school students be exempt from physical education requirements for one grading period or longer for participation in other school related activities? (YES/NO)	0.613 – 1.08	0.80/ 0.75	49	0.125 (0.258)	49	0.102 (0.228)	47	−0.032 (0.219)
Require protective gear (3)	Has your state adopted a policy requiring that students wear appropriate protective gear when engaged in physical activities during physical education? (YES/NO)	0.68 – 1.06	0.87/ 0.73	49	0.242 (0.374)	51	0.252 (0.334)	49	−0.007 (0.386)
Maintain or inspect PA facilities (3)	Has your state adopted a policy on the inspection or maintenance of playground facilities and equipment, such as playing surfaces, benches, monkey bars, and swings? (YES/NO)	0.844 – 1.03	0.89/ 0.89	49	0.565 (0.452)	46	0.464 (0.458)	45	−0.089 (0.649)
Provide PE information or materials (5)	During the past 2 years, has your state education agency provided the following information or materials for senior high school physical education: Lesson plans or learning activities for physical education? (YES/NO)	0.424-1.03	0.85/ 0.63	51	0.304 (0.373)	51	0.325 (0.291)	51	0.022 (0.407)
Discourage physical activity as punishment (4)	Has your state adopted a policy that prohibits schools from using physical activity (e.g. laps or push-ups) to punish students for bad behavior in physical education? (YES/NO)	0.740 – 1.0	0.80/ 0.78	46	0.184 (0.286)	51	0.382 (0.284)	46	0.190 (0.366)
Implementation of adaptive PE (5)	Has your state adopted a policy stating that schools will implement the following measures to meet the physical education needs of students with permanent physical or cognitive disabilities: Providing adaptive physical education as appropriate? (YES/NO)	0.863 – 0.991	0.90/ 0.92	46	0.728 (0.367)	48	0.861 (0.298)	44	0.092 (0.389)
Staff development opportunities (14)	During the past 2 years, has your state education agency provided any funding for or offered staff development on each of the following topics to those who teach physical education (including workshops, conferences, continuing education, graduate courses, or other in-kind service: Encouraging family involvement in physical activity? (YES/NO)	0.589-1.04	0.95/ 0.96	50	0.386 (0.382)	51	0.570 (0.390)	50	0.177 (0.455)
Standards and compliance for PE (6)	Which of the following methods does your state education agency use to improve district or school compliance with physical education standards or guidelines: Submission of written reports by districts or schools? (YES/NO)	0.442-0.951	0.79/ 0.80	51	0.633 (0.324)	51	0.682 (0.293)	51	0.049 (0.328)
Testing requirements for PE (6)	Does your state education agency require or recommend that senior high schools test students’ fitness levels? (Require, Recommend, Neither)	0.637 – 0.997	0.78/ 0.86	51	0.178 (0.222)	51	0.188 (0.209)	51	0.01 (0.237)
Goals and objectives for PE (5)	Do the goals or objectives for senior high school physical education specifically address each of the following student outcomes: Regular participation in physical activity? (YES/NO)	0.789 – 1.16	0.99/ 0.98	51	0.643 (0.474)	51	0.784 (0.398)	51	0.141 (0.54)
Physical activity promotion: faculty and staff (3)	During the past 12 months, has your district provided any funding for or sponsored each of the following services or programs for school faculty and staff: Physical activity and fitness counseling? (YES/NO)	0.627-0.996	NA/ 0.69	50	0.007 (0.047)	50	0.067 (0.213)	49	0.061 (0.222)
State certification for PE teachers (2)	Which of the following types of certification, licensure, or endorsement does your state offer for physical education teachers: physical education for senior high school? (YES/NO)	0.630-1.0	0.64/ 0.57	45	0.467 (0.418)	51	0.471 (0.405)	45	0.011 (0.538)
Requirement when hiring new PE teachers (2)	Has your state adopted a policy stating that newly hired staff who teach physical education at each of the following levels will have undergraduate or graduate training in physical education or a related field: Senior high school? (YES/NO)	0.75 – 1.0	0.64/ 0.60	50	0.870 (0.300)	51	0.882 (0.275)	50	0.02 (0.416)
Teaching and time requirement for PE (2)	Has your state adopted a policy that senior high schools will teach physical education? (YES/NO)	0.827-1.04	0.76/ 0.62	51	0.735 (0.392)	51	0.725 (0.364)	51	−0.01 (0.291)

Cronbach’s alpha ranged from a low of 0.54 for “Exemptions from PE: religious or disability” (PD3) in 2000 to a high of 0.99 for “Goals and Objectives for PE” in 2000. About 67% of the policy domains had alpha values greater than 0.75 and all but one alpha was greater than 0.60. On average the alphas only differed slightly between years, 0.07 units, with 10 higher in 2000 and 7 higher in 2006. The largest difference between alphas at the two time points was about 0.2 units for “Exemptions from PE: religious” and “Provide PE information”. The alpha for “Physical Activity Promotion for Staff” could not be computed in 2000 because two of the three items had zero variance.

### State-level policy domain changes

A summary of policy domain changes in each state is available in Additional file 2. Most states were missing very few policy domain change scores. Twenty-five states were missing 0, seventeen missing 1, six missing 2, and two states were missing 3 policy domain change scores. Mississippi was, however, missing 8 of 17 policy domain change scores due to incomplete data from the SHPPS in 2000. On average, states increased scores in 4.94 ± 2.76 policy domains, decreased in 3.53 ± 2.03, and had no change in 7.69 ± 2.09 policy domains. In Utah, 13 of the 17 policy domain scores increased from 2000 and 2006, while Oregon, Pennsylvania, Delaware, and Nevada all saw increases in 9 policy domains. The fewest positive policy domain changes were seen in Montana (0), Ohio (0), Missouri (1), South Carolina (1) and Alabama (1).

**Figure 2:**
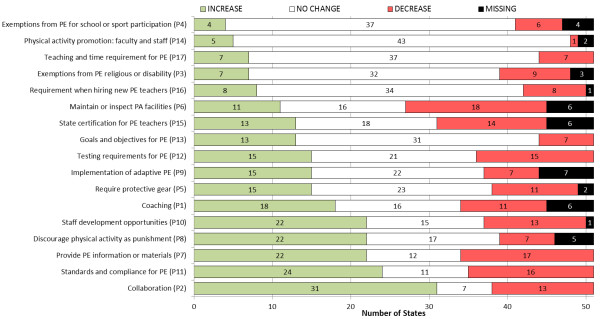
**Number of states that increased, decreased, or showed no change**^**a **^**for policy domains derived from SHPPS questionnaire.**^a^ Policy domain score was considered “No change” from 2000 to 2006 if the value changed by less than 20% of the policy domain change score standard deviation. For example, the standard deviation for change in the Collaboration factor was 0.358, so, states where the policy domain score increased or decreased by less than 0.072 (0.358*20%) were classified as “no change”. Most states in the “no change” category had policy domain change scores equal to 0. Note: For both “Exemption” factors (P3 and P4) an increase from 2000 to 2006 (in green) means more exemptions from PE allowed. While shown in green, this is considered a negative policy change.

Average policy domain change scores were positive for 12 policy domains, with the largest increases for “Discouraging PA as Punishment”, “Collaboration”, and “Staff Development Opportunities”. Using our criteria for meaningful change (at least 20% of the policy domain change score standard deviation) the average policy domain scores for only 6 policy domains changed from 2000 to 2006. Each of these policy domain scores increased overall, but did not increase in all states. In Figure 2 we show that the number of states that increased, decreased, or had no change from 2000 to 2006, varied considerably across the 17 Policy domains. For “Physical activity promotion for faculty and staff” 43 out of 49 states had no change, while 31 states showed an increase in “Collaboration”. At least 20 states also showed increases in “Provide PE information or materials” (PD7), “Staff development opportunities” (PD10), “Discourage physical activity as punishment” (PD8), and “Standards and compliance for PE” (PD11).

### Correlations among policy domains

Table 2 shows the correlations among the 17 policy domain scores for data from 2000, 2006, and among policy domain change scores. In both 2000 and 2006 the largest correlation was between “Standards” (PD11) and “Goals and Objectives” (PD13) (r ~ 0.75). In 2000, 32 correlations above 0.30 were found, 9 included “Collaboration” (PD2) making it the policy domain most related to other policy domains in 2000. In 2006, “Provide PE information and Material” (PD7) was most related to the other policy domains, being included in 8 of the 17 correlations greater than 0.30. For policy domain change scores, 10 correlations were greater than 0.30, with “Staff Development” (PD10) and “Standards” (PD11) included in 4 correlations greater than 0.30.

**Table 2:** **Correlations between policy domain change scores (change from 2000 to 2006) [*below diagonal *] and cross-sectional correlations between policy domain scores in 2000 [*above diagonal *] and in 2006 [*above diagonal; in parenthesis *] (n = 45–51)**

**Policy domains**	**P1**	**P2**	**P3**	**P4**	**P5**	**P6**	**P7**	**P8**	**P9**	**P10**	**P11**	**P12**	**P13**	**P14**	**P15**	**P16**	**P17**
1. Coaching		−0.078 (−0.005)	−0.086 (0.079)	−0.124 (0.230)	−0.127 (0.270)	0.084 (0.119)	0.110 (0.039)	0.109 (0.029)	−0.089 (0.037)	−0.049 (0.099)	0.100 (0.144)	0.216 (0.189)	0.206 (0.097)	0.065 (0.081)	0.097 (0.249)	−0.136 (0.072)	−0.025 (0.109)
2. Collaboration	−0.054		0.081 (0.172)	0.108 (0.095)	0.415 (−0.020)	0.219 (0.214)	0.352 (0.316)	0.361 (0.328)	0.262 (0.055)	0.571 (0.436)	0.631 (0.296)	0.483 (0.102)	0.481 (0.272)	0.098 (0.046)	0.169 (−0.016)	0.340 (−0.031)	0.364 (0.104)
3. Exemptions from PE religious or disability	0.026	0.172		0.183 (0.291)	0.206 (0.091)	−0.084 (0.106)	−0.106 (0.136)	−0.110 (0.004)	−0.012 (−0.050)	0.019 (0.184)	−0.145 (0.015)	−0.070 (−0.168)	−0.096 (0.049)	−0.108 (−0.001)	0.014 (0.103)	0.095 (0.276)	0.261 (0.236)
4. Exemptions from PE for sport participation	0.048	0.047	0.044		0.194 (−0.016)	−0.170 (0.070)	−0.294 (0.027)	−0.066 (−0.024)	−0.022 (0.141)	−0.213 (−0.123)	0.106 (−0.024)	−0.038 (0.004)	0.243 (0.118)	−0.073 (−0.077)	0.213 (0.292)	−0.152 (0.200)	0.090 (0.094)
5. Require protective gear	0.189	0.089	−0.128	0.248		0.125 (0.018)	0.263 (0.354)	0.088 (0.107)	0.272 (0.180)	0.374 (0.343)	0.236 (0.217)	0.083 (0.241)	0.156 (0.236)	−0.096 (0.200)	0.286 (0.105)	0.090 (0.002)	0.060 (0.195)
6. Maintain or inspect PA facilities	0.018	0.157	0.247	−0.168	0.112		0.178 (0.104)	0.018 (0.372)	0.170 (−0.209)	0.157 (0.068)	0.180 (0.176)	0.082 (0.180)	0.278 (0.260)	0.145 (0.103)	0.145 (−0.057)	−0.019 (0.146)	0.107 (0.000)
7. Provide PE information/materials	0.079	0.202	−0.092	−0.155	0.080	0.215		0.287 (0.375)	0.310 (0.311)	0.531 (0.448)	0.533 (0.372)	0.366 (0.306)	0.346 (0.294)	0.194 (0.119)	0.079 (−0.206)	0.076 (0.038)	0.302 (0.331)
8. Discourage physical activity as punishment	−0.032	0.195	0.239	−0.024	−0.119	0.104	0.286		0.173 (0.116)	0.549 (0.208)	0.304 (0.180)	0.395 (0.254)	0.232 (0.143)	0.165 (0.323)	−0.043 (0.034)	0.281 (0.267)	0.396 (0.092)
9. Implementation of adaptive PE	0.163	0.119	0.052	0.096	0.066	−0.239	0.259	0.194		0.357 (0.348)	0.472 (0.110)	0.321 (0.214)	0.364 (0.134)	0.000 (0.086)	−0.198 (0.069)	0.173 (0.190)	0.378 (0.493)
10. Staff development opportunities	0.061	0.363	0.186	−0.089	0.230	0.399	0.529	0.254	0.271		0.464 (0.067)	0.404 (0.201)	0.292 (0.044)	0.231 (0.205)	0.064 (−0.046)	0.300 (−0.141)	0.297 (0.238)
11. Standards and compliance for PE	−0.203	0.319	0.064	0.007	−0.119	−0.016	0.229	0.148	0.379	0.334		0.505 (0.394)	0.746 (0.764)	0.161 (−0.052)	−0.021 (0.026)	0.123 (0.033)	0.314 (0.063)
12. Testing requirements for PE	0.022	−0.026	−0.134	0.073	0.161	0.042	0.400	0.166	0.161	0.214	0.209		0.517 (0.281)	0.098 (0.013)	0.182 (−0.219)	0.272 (0.001)	0.236 (0.253)
13. Goals and objectives for PE	0.047	0.281	0.095	0.072	0.028	0.118	0.113	0.056	0.229	0.283	0.655	0.179		0.110 (−0.083)	0.082 (0.034)	0.033 (0.257)	0.235 (0.052)
14. PA promotion: faculty and staff	0.267	0.106	−0.250	0.117	0.174	0.147	0.099	0.152	0.200	0.039	0.079	0.047	0.107		0.194 (0.174)	0.060 (0.129)	0.099 (0.244)
15. State certification for PE teachers	0.199	0.302	0.074	0.145	0.047	0.282	−0.002	0.159	−0.138	0.120	0.003	0.189	0.162	0.207		−0.083 (0.192)	0.057 (0.046)
16. Requirement when hiring new PE teachers	0.060	0.133	0.252	0.007	0.045	0.049	−0.135	0.260	0.148	−0.092	−0.134	−0.010	0.059	0.033	0.102		0.183 (0.120)
17. Teaching and time requirement for PE	0.114	−0.037	0.321	−0.049	−0.258	−0.268	0.061	0.025	0.126	−0.068	0.023	0.062	−0.029	0.010	0.037	−0.082	

Correlations > 0.30 underlined.

## Discussion

Quantifying policy change and its impact is integral to the policy making and revision process. Building on previous work in this area, the results of this study were used to identify a set of 17 policy domains. They were developed to be specific to high-schools and to contain the same information over time, enhancing our ability to examine change in policy. Data from two administrations of the SHPPS survey (2000 and 2006), a national policy surveillance instrument, were used. The resulting policy domain scores can be applied during the evaluation process to summarize policy change related to student behavior and will be useful in gaining a better understanding of the similarities and differences among specific policies and provisions for PA and PE. In addition, it will be interesting to see how policy change progresses in each policy domain by applying these results to data from the 2012 administration of the SHPPS survey.

### State-level policy domains

Previous work in this area provided guidance in developing state-level PE and PA policy domains. In their work, Nanney and colleagues identified 10 policy domains using state-level policy and practice data for elementary, middle, junior, and senior high schools from SHPPS 2006 [6]. Nine could be applied to senior high schools (walking to school was not applicable for high schools). Of these, five are similar to those identified in the current study. Three are nearly identical (Physical Activity as Punishment (PD8), Protective Gear (PD5), and Adaptive PE (PD9)), while Testing (PD12) and Collaboration (PD2) are similar to the Assessment and Collaboration policy domains identified by Nanney et al. (2010), but contain fewer items. The difference in items is primarily due to the fact that in the previous study, items that applied to elementary, middle, and junior high school were included in the policy domain development. While some items and policy domains will be similar across grade level, we feel that grade-specific policy domain scores are useful for several reasons. First, PE requirements and implementation are quite different across grade levels. This means that while PE policies may be related for middle- and high-schools they are likely not the same. Therefore, a state-level policy domain score for “standards” that includes all grades may not truly reflect the strength or weakness in policy at a given grade level, making it more difficult to assess policy impact. Second, the available data on PE and PA participation for different aged students are often collected in different ways (e.g. High Schools collect self-report like the YRBS; elementary schools rely on observation or proxy report). This makes it difficult to compute the state-level behavioral outcomes needed for comparison to a general (all-grade levels) policy domain score. Finally, differentiation of policy effects may be particularly important during different developmental periods. For example, requiring more PE or PA in school may be most beneficial during early to middle adolescents when overall activity levels decline more rapidly, especially in girls [15]. Having only general policy domain scores would make it hard, if not impossible, to identify potentially important effects of policy change during these influential periods.

The final two policy domains identified by Nanney et al., Standards and Training, included a large number of items. In our work several smaller, more specific policy domains were identified within these larger groups of items. For example, the previous study created one training policy domain with 38 items, including 27 related to high school. Our analysis suggested that they should be separated into policy domains related to “PE certification” (PD15, PD16), “Coaches training” (PD2), and “Staff development” (PD10). Looking at our correlational and state-level change results it seems that these policy domains are distinct. For the Standards policy domain Nanney and Colleagues identified 35 items, 10 of which apply to High School. Our results suggest that these items may not represent a single policy domain, but rather, “General PE standards” (PD11), “PE goals” (PD13), and “PE teaching/time requirements” (PD17). In our correlational results, “General PE standards” and “PE Goals” had the strongest relationship (r ~ 0.75). This suggests that these policy domains might be combined. Given the other data available, like item content, scatter plots, and policy domain change scores, it is difficult to tell if these factors should be merged or if they represent separate ideas and actions that are related but need to be differentiated. At this time we suggest that these policy domains be studied separately. Future research may show that these policy domains are related to behavioral outcomes or legislative change in similar ways, but for now they should be treated as distinct.

### State-level policy domain changes

Averaged over all states, 11 of the 17 policy domain scores did not change meaningfully from 2000 to 2006. Similar information can be found in the PERSPCS data (http://class.cancer.gov/index.aspx). Their data showed that while average PE policy domain scores increased about 8%, most states (34 of 51) showed no change from 2003 to 2008. Looking at data from 2003 to 2006, dates which more closely match the SHPPS data used in this study, 43 states had zero change in PE policy domain scores. ([7]; CLASS.cancer.gov data accessed Jan 2012). While the average policy domain score results are similar, our data showed more variation between states. In our sample, every state changed on at least 4 policy domains with most having substantial change on at least 8 policy domain scores. The difference between the PERSPCS data and our results is likely related to differences in data collection and content coverage.

The PERSPCS data and scoring focus on laws and regulations in six key areas which were systematically scored by trained researchers. In contrast, SHPPS data were self-reported, and covered a greater number of policy domains and included more policy and provision items. Often, important changes in policies and provisions for PA in high schools may be implemented without specific changes to state laws and regulations. When this occurs the PERSPCS system is unlikely to detect change. It should also be noted that while one study has concluded that reliability and validity evidence for the SHPPS data is acceptable [16], measurement error could be inflating the amount of change estimated in the new policy domains. At this point it is safe to say that both scoring systems are important to understanding the relationship between policy and PA. Future research should help to pinpoint where each is most useful and how policy domain scores from each relate to behavioral outcomes.

### Limitations

This research study benefited from the comprehensiveness of the data collected in the SHPPS survey, but the number of items compared to the number of respondents was less than ideal for factor development. This is the primary reason we conducted several smaller exploratory factor analysis models and used expert judgment and inter-item relationships when making final decisions about a specific policy domain or a questionable item. With only 51 possible respondents the robustness and usefulness of some domains could be questioned. We also recognize that the correlations between combinations of policies can be influenced by unmeasured policies or other unmeasured attributes. This type of problem is not unique to this analysis, but analyses of numerous combined policies in this area of study are relatively new, and important sources of bias and confounding may not yet be fully understood. We suggest that researchers continue to search for variables that influence associations between policies and their targets and that the policy domains proposed here be reevaluated after the SHPPS survey is re-administered in 2012.

## Conclusions

Examining the effects of policy change on their intended targets is a major part of the policy evaluation-revision cycle. This research supports this type of future work by providing a means of examining changes in state-level policy domains related to PE and PA of high-school students and the faculty and staff that serve them. The results build on previous research to offer a new way to examine the effects of policy change on behaviors. Future research should to connect policy change not only to PE, but also overall PA, and to provide guidance to policy makers who seek ways to promote PA and health in children.

## Competing interests

The authors declare that they have no competing interests.

## Authors’ contributions

DH contributed to the design of study, performed statistical analysis and interpretation, and drafted the manuscript. JS contributed to the conception and design of study, interpretation of analysis, and revision of manuscript. DT contributed to the conception and design of study, interpretation of analysis, and revision of manuscript. DM contributed to the conception and design of study, advised on statistical methodology and interpretation, and revision of manuscript. Work related to this manuscript by DM was conducted prior to employment at the NIH. AR contributed to the drafting and revision of manuscript. All authors read and approved the final manuscript.

## Supplementary Material

Additional file 1 **Items from SPHHS considered in exploratory factor analysis.** This table lists all items from the 2000 and 2006 SPHHS related to physical education and physical activity and includes results from exploratory factor analysis for each item. Click here for file

Additional file 2 **Change in policy domain scores by state.** Table indicates whether a particular state had an increased (I), decreased (D), or did not change (0) for each of the 17 policy domains. Click here for file
